# Synergistic Effects of *Dysmorphococcus globosus* on Selenium Enrichment and Astaxanthin Accumulation

**DOI:** 10.3390/foods14183249

**Published:** 2025-09-18

**Authors:** Moyu Zhong, Xinxin Huang, Xinyue Zhang, Zahid Hussain, Zhaohui Zan, Qi Wang, Xiulan Xie, Maozhi Ren

**Affiliations:** 1 Functional Plant Cultivation and Application Teams, Institute of Urban Agriculture, Chinese Academy of Agricultural Sciences, Dushi Building, No. 36 Lazi East Street Road, Tianfu New Area, Chengdu 610000, China; 2 Ankang Se-Enriched Products Research and Development Center, Ankang 725000, China; 3 Chengdu National Agricultural Science and Technology Center, Chengdu 610000, China; 4 School of Agricultural Sciences, Zhengzhou University, Zhengzhou 450052, China

**Keywords:** astaxanthin, organic selenium, microalgae, hidden starvation, carotenoid, functional food

## Abstract

Hidden starvation poses a critical threat to people’s nutritional status and overall health. Developing functional agriculture can alleviate hidden starvation. This study investigates organic selenium supplementation challenges and the antioxidant potential of high-value astaxanthin. The microalgal strain *Dysmorphococcus globosus* HY13 was cultured in medium containing sodium selenite, and the effects of different sodium selenite concentrations on the growth of HY13 were analyzed. Color change was the most obvious when the medium was supplemented with 1500 mg L^−1^ selenite, with samples showing an orange-red color. The conversion efficiency of inorganic selenium to organic selenium reached 99.23%. Similarly, under selenium stress conditions, the HY13 strain accumulated high levels of astaxanthin (up to 0.86 mg g^−1^ dry weight). Thus, *D. globosus* appears to efficiently convert inorganic selenium into organic selenium and synergistically accumulate high-value astaxanthin under selenium stress, emphasizing its potential applications in functional agriculture and nutritionally fortified product development.

## 1. Introduction

Functional agriculture represents the transformation of agricultural development into a health-focused industry and involves growing products in soils or habitats that are naturally rich in beneficial components or cultivating them using biofortification and other biotechnological methods. This leads to the optimization of health-related components, such as minerals and bioactive compounds, in a standardized manner based on human needs. This is particularly important because hidden starvation is a global health concern affecting approximately 2 billion people, particularly in low- and middle-income countries [1]. Hidden nutritional deficiencies result from imbalanced nutrient intake, typically caused by high-calorie, nutrient-poor diets. Enhancing the nutritional value of agricultural products and further developing the field of functional agriculture may help to meet consumer demands related to healthy lifestyles. Mineral elements and vitamins are typically lacking during hidden starvation, and supplementation of the trace element selenium is particularly challenging [2], making it a critical nutritional gap requiring attention.

Selenium is an essential nutrient in humans that exhibits antioxidant, anti-cancer, and immune-boosting properties [3]. As the human body cannot synthesize selenium, it must be obtained from food sources [4], but the safe threshold for dietary selenium intake is within a narrow range [5]. The World Health Organization recommends a daily selenium intake of 50–200 μg d^−1^ for healthy adults, with excess and insufficient selenium intake causing critical harmful effects on human health. For example, Keshan [6] and Kashin–Beck [7] diseases, which were once prevalent in some Chinese regions, are caused by selenium deficiency. Furthermore, excessive selenium intake causes acute selenium poisoning, resulting in respiratory, gastrointestinal, or cardiovascular diseases and hypertension, and chronic selenium poisoning can lead to mental complications, hair and nail damage, and severe tooth decay and discoloration [8]. Therefore, precise selenium supplementation is crucial.

Selenium deficiency is a global concern identified in over 40 countries and regions according to the World Health Organization, and selenium content in food is closely related to geographical factors. Efforts are urgently needed to supplement daily diets with selenium. Selenium can be obtained from both organic and inorganic sources, but inorganic selenium is considered unsuitable for consumption because of its high toxicity. In contrast, organic selenium has higher bioavailability and lower toxicity [9].

Methods such as applying selenium fertilizers, crop breeding, and genetic modification are commonly used to increase the selenium content in crops. Higher plants can be selenium-enriched through biofortification; for example, rice treated with exogenous selenium fertilizers typically meets the standard to be considered selenium-enriched [10]. Plants in the Brassicaceae family also efficiently accumulate selenium. Lettuce sprouts treated with sodium selenite were found to contain 100% organic selenium [11]. However, higher plants do not require selenium for growth and only metabolize this element through sulfur pathways [12], resulting in low selenium conversion rates. Selenium biofortification relies on applying selenium to soils directly and foliar spraying of selenium fertilizers. Applying selenium to the soil results in high levels of selenium being wasted. Over 80% of selenate can only be fixed in the soil for a short period, with 80–95% of selenate potentially being lost due to irrigation or rainfall [13].

Plant uptake of selenium is not solely determined by the total selenium concentration in the soil because bioavailability is affected by the complex physicochemical properties of the soil [14]. Plants grown in low-selenium soils typically lack sufficient selenium, whereas those grown in high-selenium soils may contain toxic inorganic selenium [15]. Foliar spraying of selenium fertilizer avoids the transport of selenium from the roots and offers higher bioavailability compared to soil-based selenium application. However, vascular plants are morphologically complex making it difficult to achieve precise regulation of selenium accumulation in edible tissues [16]. Therefore, relying on conventional diets of grains, vegetables, and fruits may not result in stable, precise, and high-quality selenium intake.

Another common source of selenium is microorganisms, including bacteria and fungi. *Lactobacillus* is a selenium-enriching bacterium that plays a role in human gut health and immune regulation while exhibiting high selenium conversion efficiency, making it a promising candidate for application as a food supplement [17]. Selenium-enriched yeast is directly used as a dietary supplement or in the processing of fermented foods [18]. However, selenium-enriched microorganisms generally exhibit low selenium tolerance, with most tolerating less than 1000 mg L^−1^. For instance, the maximum selenium tolerance concentration for *Lactobacillus* is only 150 mg L^−1^. Although yeast cells can accumulate higher levels of selenium, they often store excessive amounts of inorganic selenium as a result, which is also undesirable for food-grade applications [19]. However, the organic selenium converted by plants and yeast primarily exists in the form of selenomethionine, which has low bioavailability in the human body and must be metabolized in the liver through the methionine cycle and trans-sulphuration pathway to generate selenocysteine for utilization [15].

Organic selenium primarily includes selenoamino acids, selenoproteins, selenium polysaccharides, selenium nucleic acids, and various methylated selenium compounds. Compared with other species, algae contain abundant organic selenium compounds. In microalgae, 54,541 genes related to selenium metabolism have been identified across 160 species [20]. The mechanisms underlying selenium metabolism in microalgae are complex but similar to those in humans and mammals. Genes containing SECIS elements also show sequence similarity between animals and algae, suggesting a common origin between selenoprotein synthesis in microalgae and mammals [21]. As the primary absorbers of selenium in aquatic ecosystems, microalgae rapidly absorb inorganic selenium from water and convert it into an organic form, which is efficiently absorbed by the human body, making microalgae excellent carriers for selenium biofortification. Therefore, using microalgae as a chassis organism and enhancing their organic selenium content through agronomic biofortification is considered a simple, rapid, and effective solution to address the challenge of selenium deficiency in hidden starvation.

The use of microalgae to meet nutritional needs has many advantages. Microalgae are rich in nutrients such as lipids, proteins, and carbohydrates. They exhibit higher energy utilization efficiency, stronger stress resistance, higher yields, better edibility of the entire organism, and higher nutritional value than higher plants. Additionally, they can be industrially produced without being limited by seasons or land. These characteristics have prompted the increasing application of microalgae in various fields [22].

Microalgae produce various carotenoids, which are common pigments in plants and microorganisms and possess biological activity. In humans, carotenoids exert antioxidant effects and have photoprotective characteristics [23]. They also help to combat oxidative stress by inhibiting lipid peroxidation and DNA damage. Carotenoids produced by microalgae include β-carotene, lutein, zeaxanthin, adonixanthin, canthaxanthin, β-cryptoxanthin, and astaxanthin. Astaxanthin, the final product of the carotenoid biosynthesis pathway, is the most commercially valuable because of its strong antioxidant properties.

Astaxanthin protects biomacromolecules from oxidative damage. Most natural astaxanthins, particularly those produced by microalgae and plants, exist in esterified forms such as mono- and di-esters that are more stable than their free forms. Astaxanthin reportedly has anti-aging, tumor growth inhibition, immune-boosting, and cardiovascular disease treatment effects [24,25]. Only natural astaxanthin is approved as a food ingredient because of its high safety [26]. The most commonly used microalgae for natural astaxanthin production is *Haematococcus lacustris* (formerly *Haematococcus pluvialis*), which accumulates substantial levels of astaxanthin. However, its growth rate is slow and biomass yield is low [27], and it is easily contaminated by other fast-growing organisms. Therefore, alternative algal strains are being explored.

*Dysmorphococcus globosus* is a widely distributed alga belonging to the Phacotaceae family within the class Chlorophyceae and phylum Chlorophyta. Species of this genus accumulate carotenoids and are an excellent source of natural astaxanthin, indicating their high commercial potential [28]. Laboratory studies showed that the *D. globosus* strain HY13 accumulates pigments rapidly under selenium treatment, suggesting its potential for synergistic enrichment of selenium and astaxanthin. However, relevant studies of these abilities are lacking. Therefore, this study was conducted to examine the growth physiological indicators of HY13 in terms of its concentration, growth, and products to investigate the impact of selenium on astaxanthin accumulation in HY13.

## 2. Materials and Methods

### 2.1. Sample Collection

Samples were collected between March and April 2023. The algal strain ZY54 was collected from Shuang’an Naore Village, Ziyang County, Ankang City, Shaanxi Province (108°56′27″ E, 32°66′8″ N); ZY23 from Lianfeng Village, Donghe Town, Ziyang County (108°60′3″ E, 32°45′81″ N); ZY24 from Donghe Township, Ziyang County, Qianhe Village (108°60′92″ E, 32°49′31″ N); HY13 from Zigou Village, Xuanwo Town, Hanyin County (108°40′22″ E, 32°77′83″ N); ES2 from Enshi City, Hubei Province (109°48′32″ E, 30°23′06″ N); HZ1 from West Lake District, Hangzhou City, Zhejiang Province (120°13′02″ E, 30°25′93″ N); and TF3 from Dujiangyan City, Sichuan Province (103°60′96″ E, 31°00′15″ N). During sampling, we located greenish turbid water bodies in selenium-rich areas, within which we used a phytoplankton net with a 64 μm aperture (200 mesh) submerged 50 cm to collect water samples in 50 mL centrifuge tubes at random positions. The samples were maintained at 2–8 °C throughout transportation, and then 15 mL of sample was transferred into a new centrifuge tube and allowed to settle at room temperature for 1–2 h. The majority of the supernatant was gently removed while retaining 2–3 mL of liquid at the bottom of the tube. After mixing, samples were placed in a shake incubator at 25 °C and 0.6540 × *g* for 6 h to fully activate them for subsequent algal separation.

### 2.2. Isolation and Purification of Microalgae

Micromanipulation is a single-cell isolation method for capturing and separating single cells from environmental samples. Under an inverted light microscope (IX73P2F, Olympus, Tokyo, Japan), microcapillary tubes were used to separate individual cells from the cell mixture and avoid contamination from other non-target cells. The individual cells were transferred to a suitable culture environment for incubation.

Algae were purified using the plate scribing method because of their sparse distribution in the collected water samples, eliminating the need for dilution. Subsequently, 50 μL of the water samples was directly collected and spread on plates containing BG11 solid medium for blue-green algae (Qingdao Hi-Tech Industrial Park HaiBo Biotechnology Co., Ltd., Qingdao, China) with 1.5 g L^−1^ sodium acetate as a carbon source. Then, static culture under uninterrupted light for approximately 15 days at a constant temperature of 25 °C and light intensity of 50 μmol m^−2^ s^−1^ was carried out. Algae with different morphologies and colors were collected from single colonies, and the cells were cultured in a suitable culture environment and identified.

### 2.3. Identification of Microalgae

Purified single algal colonies were inoculated in liquid medium and cultured for 7 days to the logarithmic growth stage. The algal solution was centrifuged at 11,180× *g* and 25 °C for 10 min, and approximately 100 mg of fresh weight was collected. Microalgal genomic DNA was extracted using a DP360 Polysaccharide and Polyphenol Plant Genomic DNA Extraction Kit (Tiangen Biochemical Technology Co., Ltd., Beijing, China), and the extracted DNA samples were subjected to PCR using Q5 high-fidelity polymerase (New England Biolabs, Ipswich, MA, USA). The obtained DNA samples were amplified by PCR using specific primers *(18S* ribosomal RNA gene, *18S rRNA*-F: 5′-AACCTGGTTGATCCTGCCAGT-3′, *18S rRNA*-R: 5′-TGATCCTTCTGCAGGTTCACCTAC-3′; photosystem I P700 chlorophyll *a* apoprotein A2, *psaB*-F: 5′-GGTGGTTTTCATCCACAAACTC-3′, *psaB*-R: 5′-GAACCACGTGCATCTAAAGCACCT-3′) [29]. These primers targeted different regions of the microalgal genome for comprehensive analysis. The PCR program was as follows: 98 °C for 30 s; followed by denaturation at 98 °C for 10 s, annealing at 55 °C for 30 s, extension at 72 °C for 1 min for 35 cycles; and termination at 72 °C for 2 min. The PCR products were electrophoresed (164–5050, Bio-Rad Laboratories, Hercules, CA, USA) on a 1.5% agarose gel at 220 V for 25 min. After electrophoresis, the DNA was sequenced by Sangon Biotech Co., Ltd. (Shanghai, China). The sequences were uploaded to the National Center for Biotechnology Information database for homology comparison, and sequences with high homology were downloaded [30]. MEGA 5.05 software was used to construct a phylogenetic tree using the neighbor-joining method [31,32].

Microscopic changes in the algal bodies were observed using an optical microscope connected to a computer, and the cells were imaged in real-time using cellSens Dimension Software (Olympus) system (Tokyo, Japan). After adjustment to the appropriate magnification, the software displayed the mobile state and structural characteristics of the microalgal cells and photographed them.

The submicroscopic structure of the microalgal cells was observed using a transmission electron microscope (JEM-1400-FLASH, JEOL, Tokyo, Japan). Cells were pre-fixed with 3% glutaraldehyde, post-fixed with 1% osmium tetroxide, dehydrated in a series of graded acetone solutions, and embedded in paraffin. Semi-thin sections were stained with methylene blue, and ultrathin sections were cut with a diamond knife and stained with uranyl acetate and lead citrate.

Microalgal cell surfaces were visualized using a scanning electron microscope (Apreo 2 S, FEI, Hillsboro, OR, USA). Sample precipitates were collected through centrifugation, rinsed gently with phosphate-buffered saline after which the phosphate-buffered saline was discarded, and fixed in suspension by adding 3% glutaraldehyde. The samples were washed three times with ultrapure water for 10 min each time, followed by fixation in 1% osmium acid for 1–2 h and three washes (for 10 min each time) with ultrapure water. These samples were dehydrated through a graded alcohol series with concentrations of 30%, 50%, 70%, 90%, and 100% via gradual replacement from low to high, and the 100% concentration was changed three times (15 min each time). The samples were dropped onto coverslips and placed in a critical point desiccator for drying. Coverslips were glued onto the sample stage with conductive adhesive and placed in an ion-sputtering instrument for gold spraying. Subsequently, the samples were glued with conductive adhesive onto the sample stage, and the region of interest was observed under a scanning electron microscope.

### 2.4. Optimization of Carbon Source in Culture Medium

**Figure A1:**
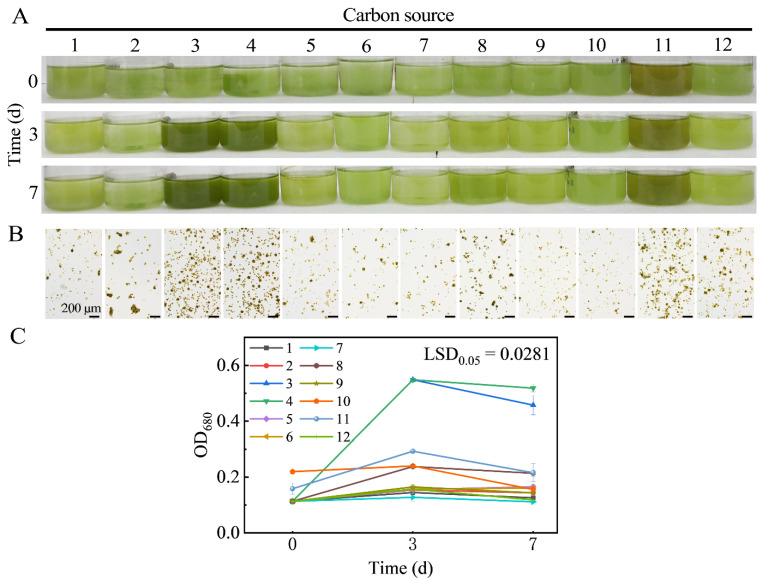
Carbon source screening results. (**A**): Color change of Dysmorphococcus *globosus* HY13 after treatment with different carbon sources for 7 days. (**B**): Effects of treatment with different carbon sources on HY13 cell density. The scale in all cell maps is 200 μm. (**C**): Optical density changes at 680 nm of HY13 between days 0 and 7 after treatment with different carbon sources. Each data point represents the mean of three replicates; error bars indicate the standard error of the mean. The numbers representing carbon sources indicate the following: 1, sodium carbonate; 2, sodium bicarbonate; 3, acetic acid; 4, sodium acetate; 5, glycerol; 6, malic acid; 7, xylitol; 8, glucose; 9, sucrose; 10, corn starch; 11, molasses; 12, blank control.

The cultivation conditions for microalgal strain HY13 were optimized based on nutritional and non-nutritional factors. As the nutritional factor, the carbon source in the culture medium was screened. Based on the number of carbon atoms in a molecule, sodium carbonate, sodium bicarbonate, acetic acid, sodium acetate, glycerol, malic acid, xylitol, glucose, sucrose, corn starch, and molasses were selected as carbon sources for microalgal culture media. Considering the characteristics of various microalgal strains, a carbon content of 100 mmol L^−1^ was selected as the standard and added to basic BG11 medium (Figure A1). All experiments were conducted with three biological replicates (n = 3). Comparison of the results indicated that sodium acetate was the most efficiently utilized carbon source by *D. globosus* strain HY13.

As non-nutritional conditions, the temperature, rotation speed, and light conditions for microalgal cultivation were screened. We found that the microalgal strain should be cultured in a shaker (ZQZY-AGF8E, Zhichu, Shanghai, China) at 0.6540× *g*, a constant temperature of 25 °C, and under continuous light at 600 μmol m^−2^ s^−1^. A plant light analyzer (PLA-30, EverFINE, Hangzhou, China) was used to measure the light intensity.

### 2.5. Screening of Selenium-Tolerant Microalgal Strains

Microalgal strains ZY23, ZY24, ZY35, ZY53, TF3, ES2, HZ1, and HY13, which were cultured in test medium for 7 days until reaching the logarithmic growth phase, were allowed to settle naturally. The supernatant was removed, and the algae were transferred into fresh basic medium. The initial microalgal growth density was adjusted to 0.5 based on measurements of the optical density at 680 nm (OD_680_) with a full-wavelength scanning multifunctional reader (Spark, TECAN, Männedorf, Switzerland). Microalgal suspensions of 50 mL were aliquoted into 100 mL conical flasks for selenium treatments. The treatment was repeated three times (n = 3).

We employed high concentrations of selenite treatment to screen for selenium-tolerant microalgal strains. Sodium selenite was added at 2000 mg L^−1^ to the culture medium of different microalgal strains. The microalgal suspensions were incubated at 25 °C with a rotation speed of 0.6540× *g* and under continuous light with an intensity of 600 μmol m^−2^ s^−1^ for 7 days, during which the state of the microalgal strains was observed. The macroscopic color of the microalgal strains and changes in the properties of the microalgal suspensions were recorded daily using a camera (EW-83M, Canon, Tokyo, Japan). After 7 days, the microalgal suspensions were examined under an inverted optical microscope to capture images; 200 μL of the microalgal suspension was collected and placed in a 96-well plate, and the OD_680_ of the microalgal strains was measured using a microplate reader. Additionally, 200 μL of the microalgal suspension was placed in a black 96-well plate, and the maximum photochemical efficiency (*F*v/*F*m) and quantum efficiency (*Y*(II)) of photosystem II (PSII) in the microalgal suspension were determined using a chlorophyll fluorometer Maxi IMAGING-PAM (WALZ, Effeltrich, Germany), with the *F*v/*F*m obtained using ImagingWin v2.56p software. As chlorophyll content and cellular activity are positively correlated, chlorophyll fluorescence detection can also reflect cell vitality. Damage to PSII is often the first manifestation of stress in plant and algal cells, particularly under selenium stress [33]. Selenium can interfere with the light-driven electron transport from PSII to PSI [34]. All measurements were performed with three technical replicates (n = 3).

### 2.6. Establishment of a Selenium-Enriched Microalgae Culture System

Microalgae strain HY13, which was cultured in BG11 basal medium for 7 days to logarithmic phase, was left to settle naturally. The supernatant was transferred into fresh BG11 basal medium. The OD_680_ of the initial microalgal concentration was adjusted to 0.5, and 50 mL of microalgal sap was dispensed into 100 mL conical flasks for selenium treatments. All replicates in our experiments were derived from the same algal strain, HY13, and samples from the same treatment group were aliquoted from the same culture flask.

Regulations for microalgae usage vary by region. Nonetheless, excessive addition of microalgae can affect the texture and taste of food products, and even small amounts can influence product composition and properties. Therefore, current microalgae-containing products generally only contain low levels. For microalgae with a long history of consumption, such as *Chlorella* and *Spirulina*, the addition level in foods like bread and biscuits typically does not exceed 10% [35]. Héctor Hernández et al. summarized cases of microalgae incorporation in ice cream, in which the content remained below 0.5% [36]. Furthermore, the United States Food and Drug Administration [37] has established limits for microalgae usage in food: 100 mg kg^−1^ *Dunaliella salina* in all products (GRN No. 351), 0.15 mg *H. pluvialis* per serving (GRN No. 580), 1.35 g *Chlorella* (90% daily intake), and 500 mg *Euglena gracilis* per serving (GRN No. 697). Considering the amount of microalgae in the product, we chose to add a high dose of sodium selenite.

To each conical flask, we added 0, 500, 1000, 1500, and 2000 mg L^−1^ sodium selenite in three replicates for each treatment. The microalgal sap was incubated at 25 °C, 0.6540× *g*, and 600 μmol m^−2^ s^−1^ for 7 days. Every other day, a camera was used to take pictures to record changes in the macro color and traits of the microalgal sap, and samples were collected to evaluate cellular changes in the microalgal strain under an inverted optical microscope. Samples were collected at 0, 1, 3, 5, and 7 days to measure OD_680_ and chlorophyll fluorescence, with both macroscopic and microscopic photographs taken at each sampling interval. To ensure randomization, each sample was manually shaken thoroughly before sampling to achieve complete mixing of the algal culture. All measurements were performed with three technical replicates (n = 3). To eliminate systematic errors caused by potential gradients in temperature, light, or slight vibration within the incubator, all replicate samples were placed on the same shaker under identical conditions and in the same position. Environmental conditions at that specific location were monitored to ensure stability.

### 2.7. Synergistic Accumulation Effects of Selenium and Astaxanthin

#### 2.7.1. Detection of Pigments Using High-Performance Liquid Chromatography

Sample pigments were extracted using the GB/T31520-2015 standard [38]. After 7 days, cultured microalgal cells were washed five times with sterile water to remove residual extracellular contents. The cleaned cells were centrifuged, and the supernatant was poured off. The microalgal cells were lyophilized using a freeze dryer (SCIENTZ-10ND, Scientz, Ningbo, China) to obtain microalgal powder. The lyophilized microalgal sample (20 mg) was weighed in a 2 mL centrifuge tube, and 0.1 mL of dichloromethane-methanol solution was added. The cell wall was broken by complete grinding using the steel ball milling method. The suspension was transferred into a 10 mL centrifuge tube, the sample in the 2 mL centrifuge tube was washed three times with 1 mL of dichloromethane-methanol solution, and the extracts were combined. The suspension was pulverized via ultrasonication for 5 min and then centrifuged at 6578× *g* and 5 °C for 5 min. The supernatant was transferred into a new 10 mL centrifuge tube, and 2 mL of methylene chloride-methanol solution was mixed with the microalgal scum. The above steps were repeated more than three times, and the supernatant was combined until the extracted microalgal scum appeared white. The supernatant was volume-stabilized with methylene chloride-methanol solution to 10 mL and left to stand for 15 min. Next, 5 mL of the supernatant was added to a new centrifuge tube, and 0.7 mL of sodium hydroxide-methanol solution was added to the supernatant, vortexed and mixed, sealed, and reacted in a refrigerator overnight for 12–14 h at 5 °C. Subsequently, 0.4 mL of 2% phosphoric acid-methanol solution was added to the reaction solution to neutralize the remaining alkali, and the mixture was filtered through a 0.45-μm membrane. The filtrate was used as the sample solution.

The pigment content was determined using high-performance liquid chromatography (HPLC) [39] on a 120EC-C18 column (150 × 3 mm, 2.7 μm). Mobile phase solution A was composed of 50% ultrapure water and 50% methanol solution containing 0.1% (*v*/*v*) formic acid, and solution B was 80% methyl tert-butyl ether and 20% methanol solution containing 0.1% (*v*/*v*) formic acid. The temperature of the column was 35 °C, detection wavelength was 475 nm, injection volume was 10 μL, and flow rate was 1 mL min^−1^. All measurements were performed with three technical replicates (n = 3).

The following analytical standards were purchased: astaxanthin (A114383 Aladdin Scientific, Shanghai, China), adonixanthin (zzstandard ZT-37002 (3S,3’R)-adonixanthin; SHANGHAI ZZBIO CO., Ltd., Shanghai, China), zeaxanthin (14681, Sigma-Aldrich, Saint Louis, MO, USA), lutein (07168, Sigma-Aldrich), canthaxanthin (11775, Sigma-Aldrich), β-cryptoxanthin (C6368, Sigma-Aldrich), and β-carotene (C4582, Sigma-Aldrich). Each standard solution was prepared at five concentration levels. The solutions were injected in order from low to high concentration. Before measuring each sample, the standard solution was used to calibrate the retention time of the standard peak, ensuring that the deviation from the analytical method was <1%.

#### 2.7.2. Detection of Selenium Content and Morphology

The total selenium and inorganic selenium contents in the samples were determined using the first method of the GB5009.93-2017 standard [40]. Freeze-dried microalgal powder (0.5 g) was placed in a conical flask; 10 mL of nitric acid-perchloric acid mixture (9:1) and a few glass beads were added, and the dish was covered for cold digestion overnight. On the following day, the solution was heated on a hot plate, and nitric acid was added until the solution was clear and colorless with white smoke. Heating was continued until the remaining volume was approximately 2 mL. After cooling, 5 mL of 6 mol L^−1^ hydrochloric acid solution was added, and heating was continued until the solution was clear and colorless with white smoke. After cooling, 10 mL of the solution was transferred into a volumetric flask, 100 g L^−1^ potassium ferrocyanide solution and 2.5 mL water were added, and the sample was mixed before measurement. The sample solution was introduced into an atomic fluorescence photometer (YQ-ZJ-007, Thermo Fisher Scientific, Waltham, MA, USA) to determine the fluorescence intensity, which was compared with a standard curve for quantification. The reference standards used were from the National Sharing Platform for Reference Materials: Selenate in Water (GBW10033), Selenite in Water (GBW10032), and Solution of Se Standard (GBW(E)080215).

The inorganic selenium content was measured. The selenium form was detected through inductively coupled plasma-mass spectrometry. Freeze-dried samples (10 μg) were weighed and dissolved in 10 mL of ultrapure water, followed by grinding to obtain a homogenate. The homogenate was centrifuged at 925× *g* and 20 °C for 10 min to obtain the supernatant. Next, cyclohexane (gas chromatography-grade, 10 mL) was added, followed by vortex mixing. The liquid was allowed to partition for 10 min, and the aqueous phase was collected. The aqueous phase was acidified with 15% hydrochloric acid and filtered through a 0.22-µm filter. Samples were analyzed using an inductively coupled plasma mass spectrometer (Orbitrap Q-Exactive HF mass spectrograph, Thermo Fisher Scientific). The total selenium content was subtracted from the inorganic selenium content to infer the organic selenium content [41]. All measurements were performed with three technical replicates (n = 3).

### 2.8. Data Processing

Data are presented as the mean ± standard error of the mean (n = 3), and standard errors were calculated using Excel software. The statistical significance of differences between means was analyzed using Duncan’s least significant difference (LSD) test. LSD_0.05_ is a basic concept in statistics for comparing means of multiple groups and is common in various comparisons after analysis of variance. If the difference between the means of two groups was more significant than the LSD_0.05_, the two groups were considered statistically significantly different. The LSD_0.05_ was calculated using the DPS Data Processing System version 9.01 (DPS, Hangzhou, China). Comparisons were made to determine whether the differences were significant based on the results of the LSD_0.05_ calculations. Statistical analysis graphs were plotted using Origin 2024 software (OriginLab, Northampton, MA, USA).

## 3. Results and Discussion

### 3.1. Isolation, Purification, and Identification of Microalgae

Dispersed water samples were collected from the same water body in selenium-enriched areas using an instantaneous sampling method at random multiple points, and microalgal strains were isolated using micromanipulation and plate delineation methods. Eight microalgal strains were isolated from seven areas.

#### 3.1.1. Morphological Characterization of Microalgae

**Figure 1:**
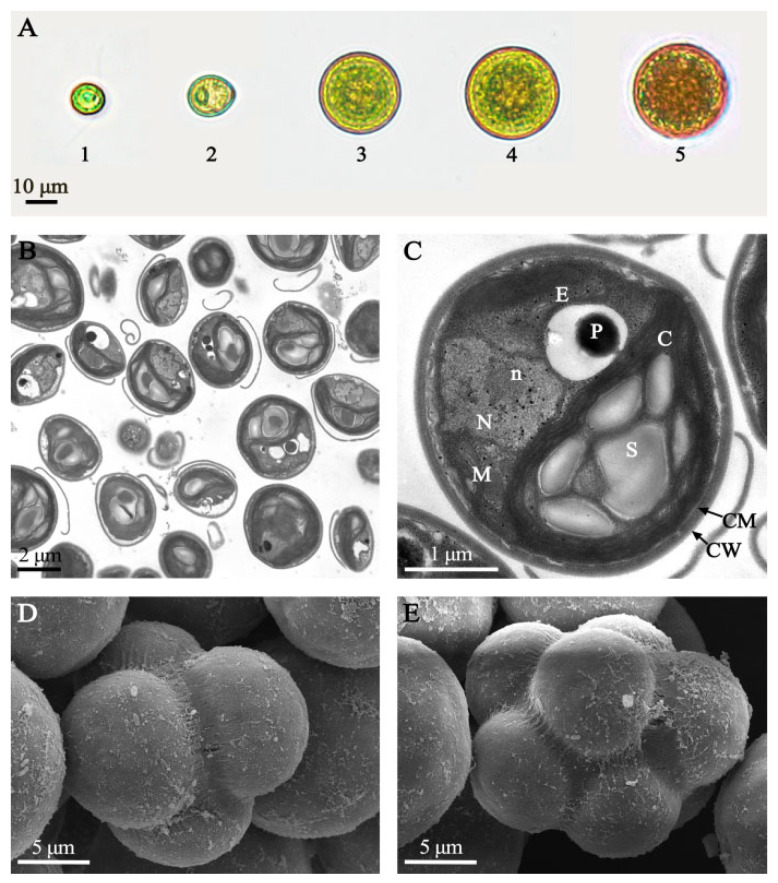
Morphological analysis of the new microalgal strain *Dysmorphococcus globosus* HY13. (**A**): Morphological changes during growth of *D. globosus* HY13. A1: Motile cell under vegetative growth conditions (day 1); A2: Initially immotile cell under vegetative growth conditions (day 2); A3: Medium-term immotile cell under vegetative growth conditions (day 3); A4: Mature cell under vegetative growth conditions (day 5); A5: Immotile cell under inductive growth conditions (day 7); (**B**,**C**): Transmission electron microscopy images of HY13. C, chloroplast; CM, cell membrane; CW, cell wall; M, mitochondria; N, nucleus; n: nucleolus; P, pyrenoid; S, starch; E: endoplasmic reticulum; (**D**,**E**): Scanning electron microscopy images of HY13.

The morphological characteristics of microalgal strain HY13 were observed using a light microscope. The cell diameter was 10–40 μm, and the complete life history comprised several stages: the initial stage was a swimming spore, which relied on the two equal-length flagella at the top of the cell for locomotion, and the cell appeared green; the flagella were shed, cell was spherical, cytoplasm gradually deepened in color, and pigment was accumulated to confer an orange-red color (Figure 1A).

The submicroscopic structure of the cell was observed using a transmission electron microscope. The primary structure of the cell is displayed in Figure 1B,C. The cell was characterized by the presence of many common structures in algae and a protein nucleus, ranging 500–800 nm in diameter. Only one protein nucleus was in each cell and was surrounded by amyloses. The chloroplast zone occupied half of the cell volume, and lamellipodia-like vesicles surrounded the starch granules in a ring. Mitochondria were present in the cytoplasm. The transverse view also showed the nucleus and nucleolus. The cell membrane was wrapped by a thick cell wall. The cell surface was viewed using a scanning electron microscope, and the processes of HY13 tetrad (Figure 1D) and octad (Figure 1E) formation were observed.

#### 3.1.2. Molecular Biological Characterization of Microalgae

DNA barcoding is a widely recognized species identification technique that compares conserved DNA region sequences of samples with those in reference databases of target species. This approach avoids the influence of morphological variations across different life stages of organisms, enabling rapid and reliable taxonomic classification at the molecular level. The *18S rDNA*, a ribosomal RNA gene with strong evolutionary conservation, is one of the target sequences for molecular identification of microalgae in the phylum Chlorophyta. Research by Ballesteros et al. demonstrated that the 18S V4 hypervariable region (300 bp) is sufficient to distinguish different isolates; however, the inclusion of a second gene remains necessary to ensure result accuracy [42]. The *psaB* gene, a core component of the photosystem I complex in microalgae, interacts with *psaA* to bind all cofactors in the electron transport chain, critically influencing light energy conversion efficiency. Due to its high conservation in green algae, we selected *psaB* as an additional genetic marker for species authentication [43].

**Figure 2:**
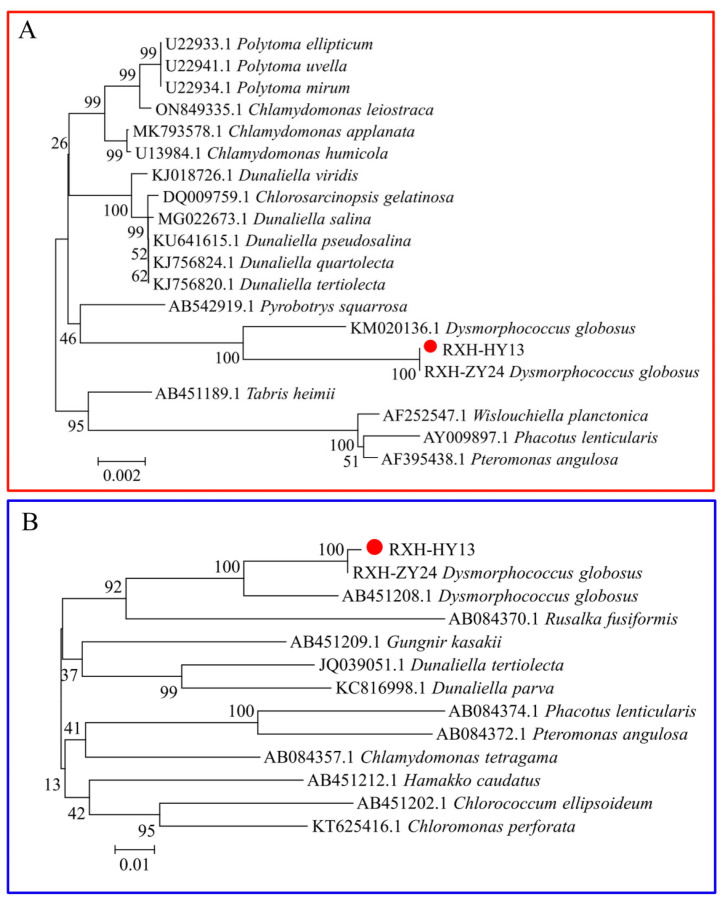
Phylogenetic trees of the isolated microalgal strains showing relatedness of their *18S rRNA* (**A**) and *psaB* (**B**) to previously obtained sequences of other algal species. The branch lengths are proportional to the evolutionary distances. Bootstrap values (>50%) from the bootstrap test (1000 replicates) are shown next to the phylogenetic trees. The scale bar shows the number of substitutions for each locus in multiple comparisons.

After nucleotide BLAST analysis, two nucleotide sequences, *18S rRNA* and *psaB*, were obtained from the isolated microalgae HY13. The extracted sequences were submitted to the National Center for Biotechnology Information for comparison with existing sequences. The accession numbers of the *18S rRNA* and *psaB* sequences were PQ632470.1 and PQ046780.1, respectively. A phylogenetic tree was constructed using the neighbor-joining method in MEGA 5.05 software (Figure 2), which indicated that the microalgal strain HY13 was similar to the sequenced microalgal strain *D. globosus*. Therefore, molecular biology identification revealed that microalgal strain HY13 was *D. globosus*.

### 3.2. Comparison of Selenium Tolerance of D. globosus from Different Habitat Sources

**Figure A2:**
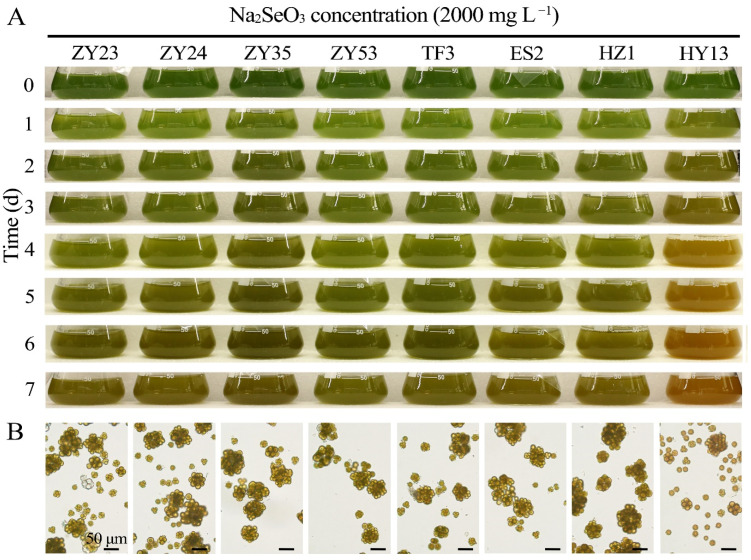
Selenium screening of isolated microalgae. (**A**): Color change in isolated microalgae culture medium after treatment with 2000 mg L^−1^ Na_2_SeO_3_. (**B**): Effects of treatment with 2000 mg L^−1^ Na_2_SeO_3_ on isolated microalgae cells. The scale in all cell maps is 50 μm.

The eight isolated microalgal strains, ZY23, ZY24, ZY35, ZY53, TF3, ES2, HZ1, and HY13, were all identified as *D. globosus*. We incubated *D. globosus* from diverse sources in medium supplemented with 2000 mg L^−1^ Na_2_SeO_3_ for 7 days to observe their traits. Among them, HY13 showed the most notable color change within 7 days and began to exhibit significantly different growth from those of the other microalgal strain on day 4, suggesting its potential to produce carotenoids in response to stress more quickly than the other strains (Figure A2).

**Figure A3:**
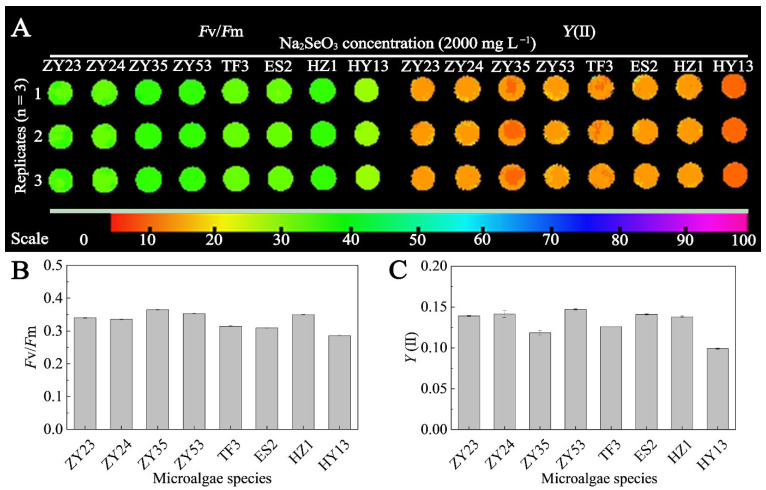
Effects of chlorophyll fluorescence parameters in isolated microalgae cultivated in the presence of 2000 mg L^−1^ Na_2_SeO_3_ for 7 days. (**A**): Phenotype of photosystem II (PSII) photochemistry. The color scale represents chlorophyll fluorescence intensity ranging from 0 to 100 (arbitrary units). (**B**): Maximum quantum efficiency of PSII photochemistry. (**C**): Actual quantum yield of PSII photochemistry. Each data point represents the mean of three replicates; error bars indicate the standard error of the mean. Significance of differences between means was analyzed using Duncan’s least significant difference test. *F*v/*F*m is the parameter associated with the maximum photochemical quantum yield of PSII; *Y*(II) is an important parameter for measuring the efficiency of conversion of light energy to chemical energy in plant photosynthesis.

**Figure A4:**
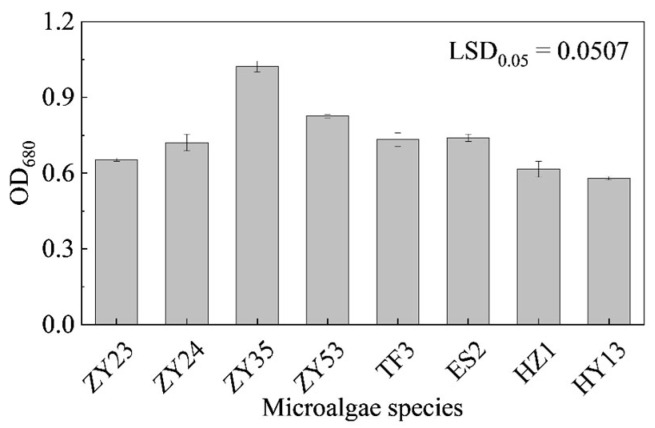
Changes in optical density at 680 nm of isolated microalgae treated with 2000 mg L^−1^ Na_2_SeO_3_. Each data point represents the mean of three replicates; error bars indicate the standard error of the mean. Significance of differences between means was analyzed using Duncan’s least significant difference test.

The maximum *F*v/*F*m and *Y*(II) of HY13 were smallest among all microalgal strains, as determined based on chlorophyll fluorescence (Figure A3), indicating that selenium had the most pronounced inhibitory effect on photosynthesis in HY13, with more chlorophyll breakdown. The OD_680_ values of the day 7 microalgal saps were also measured (Figure A4), and the densest microalgal strain was ZY35, with an OD_680_ of 1.0220, whereas HY13 had an OD_680_ of 0.5805. The differences between the mean OD_680_ values were analyzed using Duncan’s LSD test, and all microalgal strains except for HY13 and HZ1 showed significant differences. These results indicate that HY13 was significantly stressed. HY13 was chosen as the experimental microalgal strain.

### 3.3. Effects of Different Concentrations of Na_2_SeO_3_ on Astaxanthin and Selenium Enrichment of HY13

#### 3.3.1. Effects of Different Concentrations of Na_2_SeO_3_ Treatment on the Growth of HY13

Organisms absorb selenate and selenite; however, inorganic selenium exhibits toxicity in a concentration-dependent manner. Therefore, a certain concentration of selenium can promote the growth of organisms [44].

**Figure 3:**
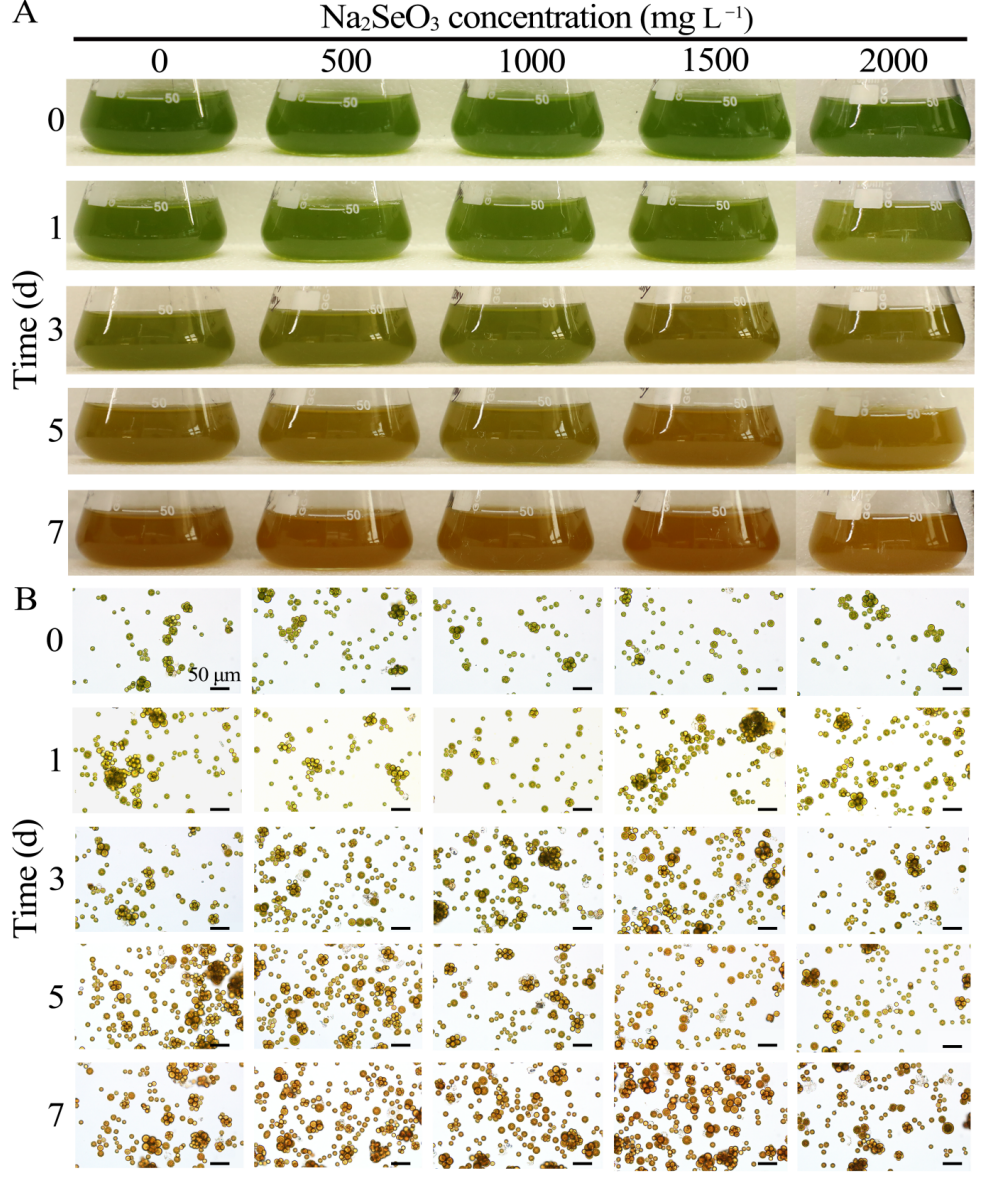
Phenotype of Na_2_SeO_3_-stressed microalgae strain HY13 during 7 days of treatment with different Na_2_SeO_3_ concentrations. (**A**): Color change in HY13 culture medium after treatment with different Na_2_SeO_3_ concentrations. (**B**): Effects of treatment with different Na_2_SeO_3_ concentrations on HY13 cells. The scale in all cell maps is 50 μm.

Microalgae produce carotenoid pigments with antioxidant properties under selenium stress at certain concentrations [45]. *D. globosus* produces antioxidant pigments, and using a high selenium concentration to create a stressful environment affected the growth of the microalgal strain. The addition of selenite to the culture medium affected the color of the microalgal strain (Figure 3A). The macroscopic observations were generally consistent with the microscopic analysis (Figure 3B). As the sodium selenite concentration increased, the orange-red coloration of the algal biomass became more pronounced. However, further analysis is required to determine the changes in pigment content. Under optical microscopy, the cells exhibited smooth and intact boundaries without any leakage of cellular contents.

**Figure A5:**
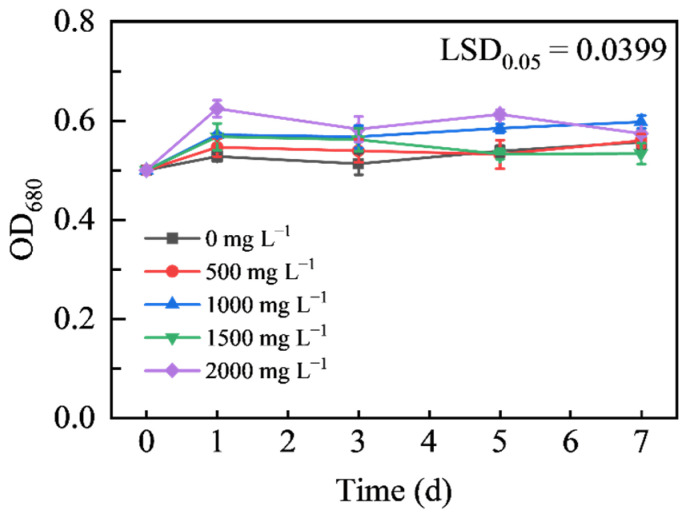
Optical density changes at 680 nm of *Dysmorphococcus globosus* HY13 between days 0 and 7 after treatment with different Na_2_SeO_3_ concentrations. Each data point represents the mean of three replicates; error bars indicate the standard error of the mean.

Changes that occurred in the presence of 0, 500, 1000, and 1500 mg L^−1^ sodium selenite were not evident within 7 days and only slightly differed between concentration groups (Figure A5). Differences between the mean OD_680_ values were analyzed using Duncan’s LSD test, with an LSD_0.05_ of 0.0399, all of which showed significantly different OD_680_ values only between days 1 and 7. The HY13 concentration in the medium supplemented with 2000 mg L^−1^ sodium selenite varied more significantly, with a slightly increased concentration on day 1 and decreased concentration on day 3, and showed an increasing trend thereafter; the OD_680_ peaked (0.6740) on day 7, showing a value of 0.1176 compared with 0.5564 on day 1.

#### 3.3.2. Effects of Different Concentrations of Na_2_SeO_3_ Treatment on Chlorophyll Fluorescence of HY13

**Figure 4:**
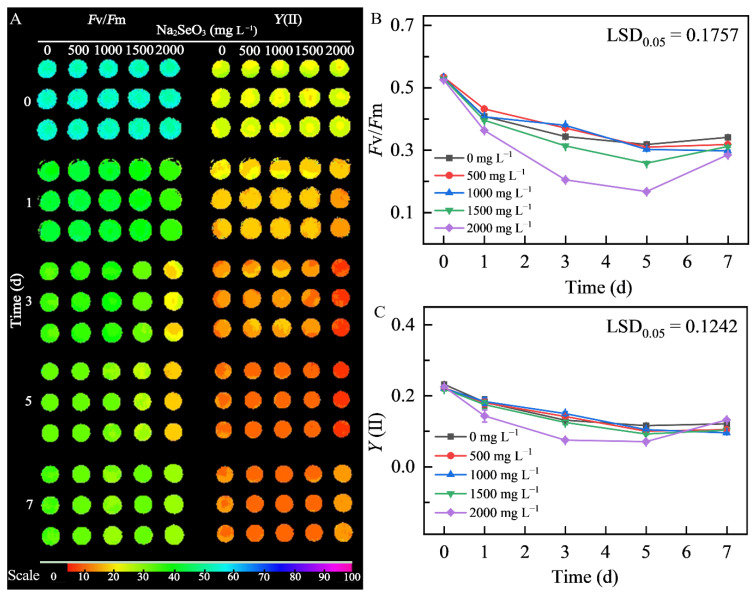
Changes in chlorophyll fluorescence parameters in microalgal strain HY13 cultivated in the presence of different Na_2_SeO_3_ concentrations for 7 days. (**A**): Phenotype of photosystem II (PSII) photochemistry. The color scale represents chlorophyll fluorescence intensity ranging from 0 to 100 (arbitrary units). (**B**): Maximum quantum efficiency. (**C**): Actual quantum yield of PSII photochemistry. Each data point represents the mean of three replicates; error bars indicate the standard error of the mean. Significance of differences between the means was analyzed using Duncan’s least significant difference (LSD) test. *F*v/*F*m is the parameter associated with the maximum photochemical quantum yield of PSII; *Y*(II) is an important parameter for measuring the efficiency of conversion of light energy to chemical energy in plant photosynthesis.

Measurement of chlorophyll fluorescence (Figure 4) indicated that all treatments decreased microalgal growth status due to the strong light; particularly, the microalgal strain treated with 2000 mg L^−1^ sodium selenite showed the most significant decreases in *F*v/*F*m and *Y*(II). By day 5, the *F*v/*F*m decreased from 0.5257 to 0.167 and *Y*(II) decreased from 0.2253 to 0.0703. The other microalgal strains showed similar changes in their growth status, with a decreasing trend in the first 5 days. However, they showed a slight rebound on day 7, possibly because of adaptation to the selenium-added environment and new pigment production.

#### 3.3.3. Effects of Different Concentrations of Na_2_SeO_3_ on Carotenoid Accumulation in HY13

Carotenoids are among the most diverse groups of pigments produced by all photosynthetic organisms; more than 600 compounds that occur naturally have been identified, including 50 in algae [46,47]. They include astaxanthin, adonixanthin, zeaxanthin, lutein, canthaxanthin, β-cryptoxanthin, and β-carotene [48]. Carotenoids can be interconverted within cells, and the biochemical and molecular aspects of carotenoid biosynthesis in higher plants are well-understood; however, the carotenoid conversion pathway in microalgae has not been widely investigated. The metabolic pathway of carotenoid biosynthesis in microalgae is similar to that in higher plants, comprising desaturation, cyclization, hydroxylation, and epoxidation to synthesize the final product [49]. Octahydro lycopene, the first colorless carotenoid in the synthetic reaction, is desaturated by specific enzymes to produce all-*trans* lycopene, which is cyclized into α-carotene and β-carotene by lycopene ε-cyclase and lycopene β-cyclase. α-Carotene is then hydroxylated at its ε- and β-rings, a process primarily catalyzed by CYP97 family hydroxylases (specifically CYP97A and CYP97C), to produce lutein. Which is the end product of the β and ε branches of the carotenoid biosynthesis pathway. β-Carotene is the immediate precursor of astaxanthin biosynthesis; however, there are multiple pathways to astaxanthin, which involve a series of hydroxylation and ketonization steps. In *H. lacustris*, β-carotene is converted to canthaxanthin through two-step ketonization catalyzed by β-carotene ketolase, which subsequently undergoes two-step hydroxylation by β-carotene hydroxylase to form astaxanthin. In *Chromochlori zofingiensis*, astaxanthin is synthesized by ketonization of zeaxanthin, also using the catalytic action of β-carotene ketolase; however, its relatively low ketonization activity leads to accumulation of the intermediate adonixanthin [50].

The contribution of different stresses to the microalgal production of pigments with antioxidant functions has been extensively demonstrated. For example, sustained strong light stress induces astaxanthin production in *Rhodococcus rainieri*, which is the most common approach used for astaxanthin production [51]. Similarly, high-salt environmental stress induces the production of astaxanthin in *R. rainieri* [52]. The green alga *C. zofingiensis* also synthesizes astaxanthin in response to external stress [53].

**Figure 5:**
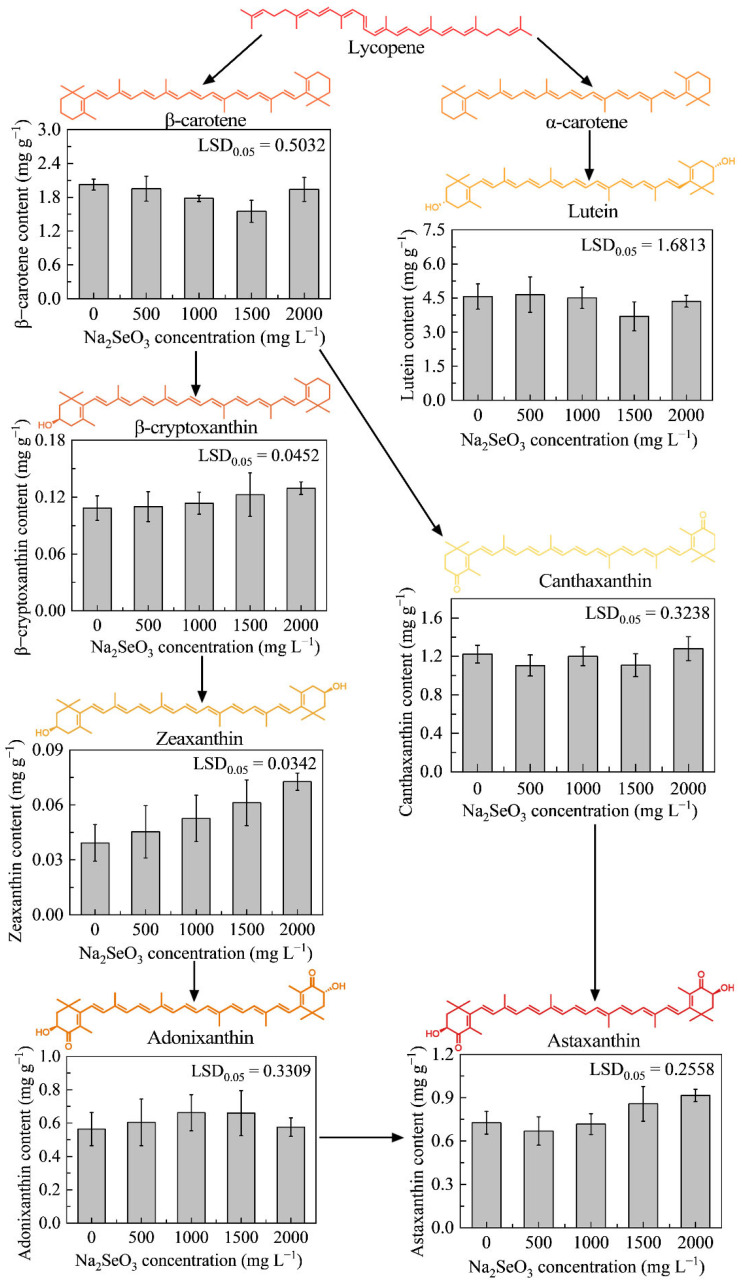
β-Carotene, lutein, β-cryptoxanthin, canthaxanthin, zeaxanthin, adonixanthin, and astaxanthin contents of strain HY13 determined on day 7 of treatment. Each data point represents the mean of three replicates; error bars indicate the standard error of the mean. Significance of differences between the means was analyzed using Duncan’s least significant difference (LSD) test.

**Figure A6:**
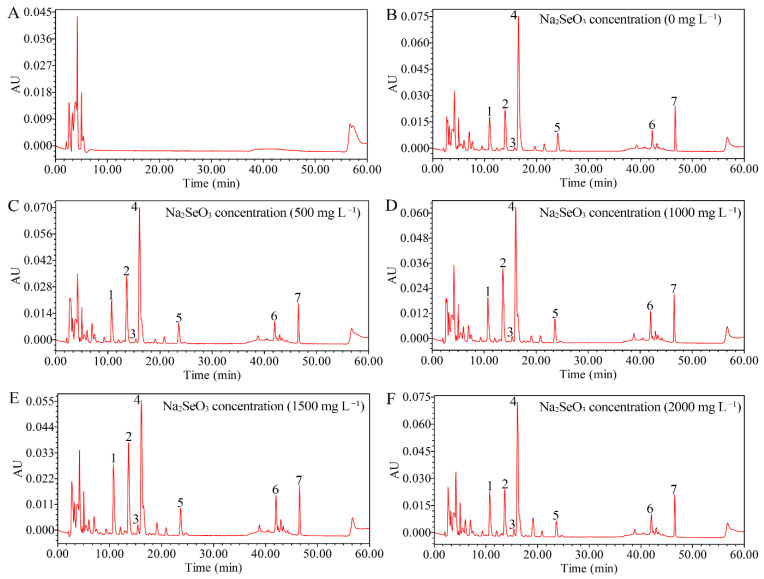
HPLC chromatograms of different Na_2_SeO_3_ concentration treatments. (**A**): Blank control group excluding the effect of reagents. (**B**–**F**): General pigment profile of *Dysmorphococcus globosus* HY13 cultivated with different Na_2_SeO_3_ concentrations for 7 days. 1, Astaxanthin; 2, adonixanthin; 3, zeaxanthin; 4, lutein; 5, canthaxanthin; 6, β-cryptoxanthin; 7, β-carotene.

**Figure A7:**
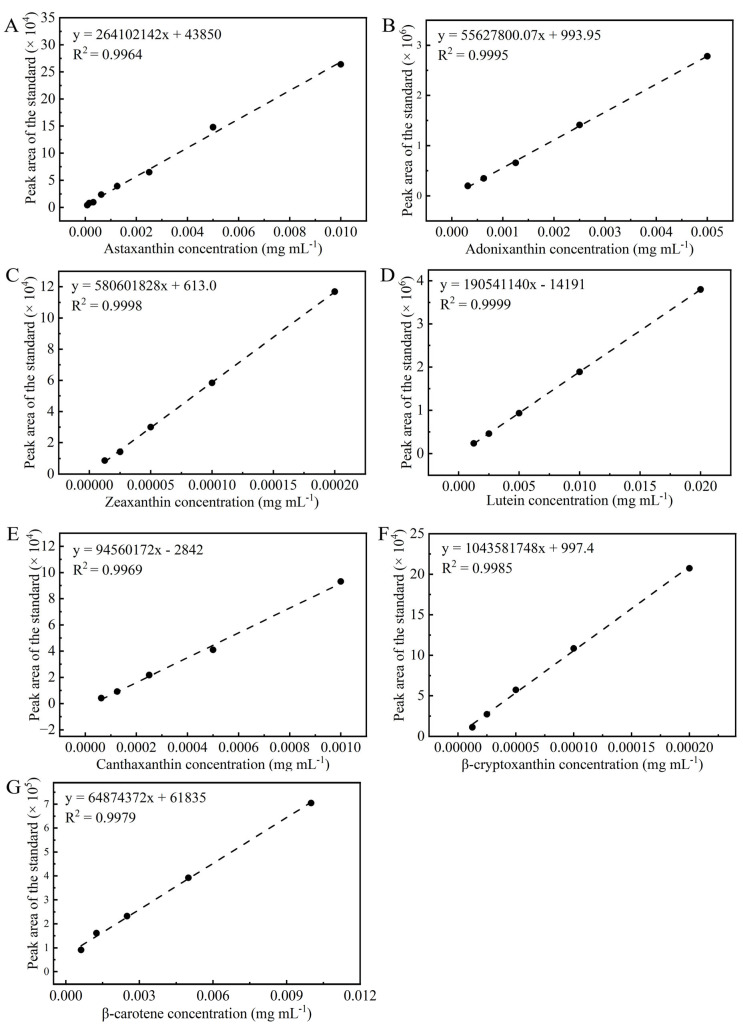
Standard curves of different types of carotenoids in HPLC: (**A**): astaxanthin; (**B**): adonixanthin; (**C**): zeaxanthin; (**D**): lutein; (**E**): canthaxanthin; (**F**): β-cryptoxanthin; and (**G**): β-carotene.

Addition of selenite ions facilitated the conversion of carotenoids from initial to final products (Figure 5 and Figure A7). The concentrations of the first few products of the conversion pathway gradually decreased with increasing sodium selenite concentrations and reached a minimum at 1500 mg L^−1^ treatment. Based on the standard curve generated from five concentration gradients of the standard solution (Figure A6), the pigment concentration in the samples was calculated. The β-carotene content was 2.0226 mg g^−1^ dry weight (DW) at 0 mg L^−1^ sodium selenite and decreased to 1.5501 mg g^−1^ DW at 1500 mg L^−1^ sodium selenite. Lutein showed the same trend, with sodium selenite treatment causing the pigment content to decrease from 4.5656 mg g^−1^ DW (at 0 mg L^−1^) to 3.6947 mg g^−1^ DW (at 1500 mg L^−1^).

The average contents of zeaxanthin, β-cryptoxanthin, and astaxanthin increased with increasing selenite ion concentrations, with the highest average contents of these three products observed at 2000 mg L^−1^ selenite, reaching 0.07264, 0.1294, and 0.92 mg g^−1^ DW, respectively. The adonixanthin contents were 0.6623 and 0.6595 mg g^−1^ DW at 1000 and 1500 mg L^−1^ selenite ion, respectively, showing very similar values. As an intermediate product of one of the pathways for astaxanthin synthesis, the canthaxanthin content did not tend to change and did not show the same or opposite trend as that of astaxanthin, suggesting that microalgal strain HY13 did not synthesize astaxanthin primarily through this pathway. Differences between the mean values of pigment content were analyzed using Duncan’s LSD test, revealing that the differences in the pigment changes were not significant.

#### 3.3.4. Effects of Different Concentrations of Na_2_SeO_3_ Treatment on the Organic Selenium Content of HY13

**Figure 6:**
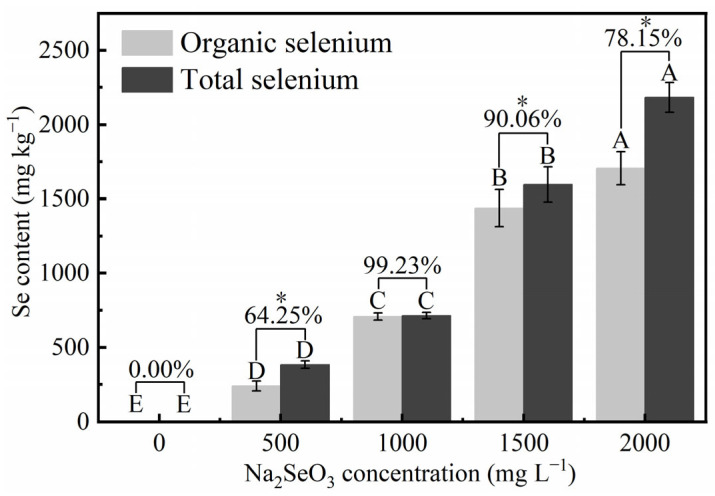
Content of different selenium forms in dried biomass and selenium biotransformation percentage of HY13 cultivated in the presence of different concentrations of Na_2_SeO_3_ for 7 days. Each data point represents the mean of three replicates; error bars indicate the standard error of the mean. The statistical significance of differences between means is indicated asterisks (* *p* < 0.05).

HY13 enriched and transformed selenium (Figure 6). At 500 mg L^−1^ sodium selenite, the total selenium content in the microalgal powder was 382.12 mg kg^−1^ and selenite ion content was 134.68 mg kg^−1^. Part of the sodium selenite was converted to sodium selenate at 1.90 mg kg^−1^, and the organic selenium content was inferred to be 245.54 mg kg^−1^ (that is, total selenium minus inorganic selenium), with a conversion efficiency of 64.25%. The total selenium content of algae cultured in 1000 mg L^−1^ sodium selenite medium was 713.43 mg kg^−1^, with a conversion efficiency of 62.33%. The total selenium content of algae cultured in 1000 mg L^−1^ sodium selenite medium was 713.43 mg kg^−1^, of which 3.85, 1.61, and 707.97 mg kg^−1^ were selenite ion, sodium selenate ion, and organic selenium, respectively, corresponding to a conversion efficiency of 99.23%. The total selenium content of the algae cultured in 1500 mg L^−1^ sodium selenite medium was 1596.30 mg kg^−1^, of which 157.03, 1.61, and 1437.66 mg kg^−1^ was selenite ion, sodium selenate ion, and organic selenium, respectively. The conversion efficiency was 90.06%. The total selenium content of algae cultured in 2000 mg L^−1^ sodium selenite medium was 2182.197 mg kg^−1^, comprising 440.90, 35.89, and 1705.40 mg kg^−1^ selenite ion, selenate ion, and organic selenium, respectively, with a conversion efficiency of 78.15%.

The conversion efficiency of HY13 from inorganic to organic selenium gradually increased with increasing sodium selenite concentrations and was optimal at 1000 mg L^−1^ sodium selenite. However, the conversion efficiency decreased with further increases in the sodium selenite concentration.

Yeasts have recently been explored for selenium enrichment. For instance, *Candida utilis* ATCC 9950 cultivated in selenium-rich agro-food industrial wastewater achieved a maximum selenium accumulation of 2.28 mg g^−1^ [54]. Although specific data on yeast-derived carotenoid species (e.g., astaxanthin, adonixanthin, and lutein) are limited, selenium is known to exert an inhibitory effect on total carotenoid production. Kieliszek et al. demonstrated that *Rhodotorula mucilaginosa* cultured in selenium-supplemented media (20 mg L^−1^ Se) reached a peak selenium content of 1.19 mg g^−1^, accompanied by significant carotenoid reduction [55]. Similarly, Sęk et al. revealed a dose-dependent decline in carotenoid levels with increasing selenium concentrations in yeast cultures [56]. However, yeasts generally display low selenium tolerance (0.5–30 mg L^−1^) and limited selenium bioaccumulation capacity, making it challenging to meet selenium enrichment standards for practical applications. Consequently, the co-enrichment of carotenoids and selenium using yeast-based systems faces inherent limitations.

Algae are excellent carriers of biological selenium, and the use of microalgae to enrich and convert selenium has been extensively studied. Pires et al. cultured *Chlorella vulgaris* in medium supplemented with 20 mg L^−1^ Na_2_SeO_4_, and 81% of the total selenium absorbed by the biomass was inferred to be converted into organic selenium [57]. Jiang et al. cultured *Spirulina platensis* in selenium-enriched medium and obtained five selenium-containing proteins with different molecular weights [58]. Compared with yeast, microalgae exhibit considerable variation in selenium tolerance. For instance, *Chlamydomonas reinhardtii* cannot survive in environments with selenium concentrations exceeding 11.85 mg L^−1^ [59]. The half-lethal concentration (LC_50_) of selenite for *H. pluvialis* is 48.032 mg L^−1^ [60]. Exposure to 100 mg L^−1^ selenate causes severe cellular damage in *Chlorella sorokiniana* [61], and sodium selenite concentrations above 500 mg L^−1^ are toxic to *S. platensis* [62].

*D. globosus* has not been extensively evaluated. Jannel et al. cultured *D. globosus* in a photobioreactor, which produced approximately 4 g L^−1^ of carotenoids, 1.2 mg g^−1^ DW of keratins, and 0.7 mg g^−1^ DW of other different forms of carotenoids, suggesting that the *D. globosus* microalgal strain is a valuable source of carotenoids [63]. Zohir et al. isolated the *D. globosus*-HI microalgal strain from Himachal Pradesh, India, and detected astaxanthin [28]. To promote astaxanthin production in a *D. globosus* microalgal strain, Zan et al. increased the Zn^2+^ concentration in the medium and found that the ZY24 strain produced the most astaxanthin (0.31 mg g^−1^ DW) in the presence of 45.5 mg L^−1^ Zn^2+^ [64].

Zheng et al. found that selenite root synergistically enriched organic selenium and astaxanthin in *R. rainieri*; however, corresponding studies in *D. globosus* are lacking [60]. We showed that a microalgal strain of *D. globosus* converted inorganic selenium into organic selenium, and the conversion efficiency increased with increasing sodium selenite concentrations. It is hypothesized that selenium stress promotes the conversion to organic selenium, which is utilized to synthesize anti-oxidative selenine and selenoproteins, preventing cellular damage. At sodium selenite concentrations of 1000–1500 mg L^−1^, the conversion efficiencies were 90–99.23%, indicating its high application potential.

## 4. Conclusions

With the improvement in living standards and widespread awareness of healthy eating, addressing selenium deficiency in the population has become an urgent issue. Firstly, market demand for selenium-enriched foods is expected to grow steadily, driving further research and development of such products. At the same time, public health interventions led by governments may also be implemented. Taking iodine as an example, China has adopted nationwide salt iodization measures to combat iodine deficiency. However, supplementation with selenium requires more cautious consideration.

Most naturally occurring selenium exists in inorganic forms, which may pose higher toxicity risks. Therefore, microalgae represent an ideal carrier for achieving low-toxicity selenium intake. Microalgae-based selenium enrichment technology can be integrated into public health strategies to develop dietary supplements targeting key populations such as children and pregnant women. This biotechnological approach offers a sustainable and precise solution that can alleviate regional selenium deficiency and improve public health.

The ability of HY13 to efficiently convert selenium while simultaneously accumulating valuable bioactive compounds offers promising avenues for the development of dual-purpose biotechnology processes that combine environmental remediation with the production of economically important metabolites. The selenium conversion efficiency of *D. globosus* HY13 reached a maximum of 99.23% at 7 days, with the sodium selenite concentration reached 1000 mg L^−1^. When the sodium selenite concentration was increased to 1500 mg L^−1^, the selenium conversion efficiency still reached 90.06%. This indicates its strong potential for application in the bioremediation of selenium-contaminated environments and the sustainable production of selenium-enriched biomass. Selenium exposure greatly influences pigment accumulation through a dual mechanism involving metabolic stimulation and stress-induced antioxidant responses. At lower concentrations, selenium promotes the synthesis of beneficial pigments without inhibiting growth or inducing significant oxidative damage, highlighting its role as a beneficial stimulant under controlled conditions [65]. In contrast, higher selenium levels trigger adaptive antioxidant mechanisms, leading to enhanced production of pigments with recognized antioxidant properties [66]. These findings underscore the strategic value of using selenium stress as a tool for enhancing the production of high-value carotenoids in microbial cultivation. Similarly, selenium stress promoted the production of various pigments with antioxidant functions by HY13, among which, astaxanthin was the most valuable high-value-added product with strong antioxidant and anti-inflammatory properties. A large amount of astaxanthin was produced at 2000 mg L^−1^ sodium selenite, reaching 0.92 mg g^−1^ DW. Following treatment with 1500 mg L^−1^ sodium selenite, the selenium conversion efficiency reached a maximum of 90.06%, whereas the astaxanthin content was 0.86 mg g^−1^ DW, which did not significantly differ from the maximum astaxanthin content. Therefore, 1500 mg L^−1^ is recommended as the optimal sodium selenite addition for selenium-enriched culture of HY13.

This study provides theoretical guidance for synergistic enrichment of selenium and astaxanthin by microalgae and offers insights into the large-scale production of selenium-enriched *D. globosus*. However, the genome of *D. globosus* has not been analyzed, and the key enzymes and pathways involved in astaxanthin synthesis require further investigation. In addition, the mechanism of organic selenium conversion is unclear, and whether *D. globosus* can synthesize selenoprotein remains unknown. Therefore, exploring related genes is essential to understanding the synthesis mechanism and internal connection between selenium and astaxanthin, as well as the mechanism by which selenium stress promotes pigment accumulation. The possibility of combining astaxanthin and selenium should be investigated to obtain products with improved application value.

## Data Availability

The original contributions presented in the study are included in the article, further inquiries can be directed to the corresponding author.

## Abbreviations

The following abbreviations are used in this manuscript:

DW	Dry weight
HPLC	High-performance liquid chromatography
LSD	Least significant difference
LC_50_	Half-lethal concentration
OD	Optical density
PSII	Photosystem II
